# Zero-shot fMRI decoding with three-dimensional registration based on diffusion tensor imaging

**DOI:** 10.1038/s41598-018-30676-3

**Published:** 2018-08-17

**Authors:** Takuya Fuchigami, Yumi Shikauchi, Ken Nakae, Manabu Shikauchi, Takeshi Ogawa, Shin Ishii

**Affiliations:** 1 Graduate School of Informatics, Kyoto University, Kyoto, 606-8501 Japan; 2 ATR Cognitive Mechanisms Laboratories, Kyoto, 619-0288 Japan; 3 Rhythm-based Brain Information Processing, RIKEN BSI-TOYOTA Collaboration Center, Wako, 351-0198 Japan; 4 Present Address: FUJIFILM Corporation, Minato, Tokyo, Japan; 5 Present Address: Cingulate Co. Ltd., Osaka, Japan

## Abstract

Functional magnetic resonance imaging (fMRI) acquisitions include a great deal of individual variability. This individuality often generates obstacles to the efficient use of databanks from multiple subjects. Although recent studies have suggested that inter-regional connectivity reflects individuality, conventional three-dimensional (3D) registration methods that calibrate inter-subject variability are based on anatomical information about the gray matter shape (e.g., T1-weighted). Here, we present a new registration method focusing more on the white matter structure, which is directly related to the connectivity in the brain, and apply it to subject-transfer brain decoding. Our registration method based on diffusion tensor imaging (DTI) transferred functional maps of each individual to a common anatomical space, where a decoding analysis of multi-voxel patterns was performed. The decoder trained on functional maps from other individuals in the common space showed a transfer decoding accuracy comparable to that of an individual decoder trained on single-subject functional maps. The DTI-based registration allowed more precise transformation of gray matter boundaries than a well-established T1-based method. These results suggest that the DTI-based registration is a promising tool for standardization of the brain functions, and moreover, will allow us to perform ‘zero-shot’ learning of decoders which is profitable in brain machine interface scenes.

## Introduction

Every individual has a unique functional brain, which is reflected by profiles of brain activity, which indicate that individuals’ patterns are as unique as fingerprints^1,2^. These individual differences sometimes prevent effective utilization of brain databanks such as in the WU-Minn Human Connectome Project (http://humanconnectome.org). One typical example of the problems is brain decoding. A brain decoder learns discrimination boundaries/regression coefficients using brain activity patterns obtained from a unique individual. Thus, the decoder is trained to the individual, and is not directly applicable to others^3,4^. The problem here is not a lack of robustness of individual decoders, but comes from the difficulty in utilizing large multi-subject databanks to construct a general decoder that works on a multitude of subjects. In general, a large number of samples present a clear advantage for finding better model parameters^5^. As studies on brain functions continue to progress, the extent of the available data and databanks containing multiple subjects increases day by day^6^. Data-driven functional analyses based on *big* imaging databases urgently require further development of sophisticated calibration methods, which are especially indispensable in the scenario of brain decoding.

According to previous studies, brain activity data measured in an additional session of several minutes helped data sharing between individuals^7,8^. Moreover, if functional images when watching a variety of visual scenes are available, multi-voxel activity patterns taken by functional magnetic resonance imaging (fMRI) can be calibrated between individuals, according to the technology called hyper-alignment^9–11^. In particular, a decoder trained on the activities of *reference* subjects was calibrated to another *target* subject using an additional dataset containing data from both the reference and target subjects. The fine-tuned decoder achieved a higher decoding accuracy with a small burden on the target subject. However, a subject-transfer decoding with *zero-shot* training, indicating that no additional *functional* images are required from the target subject, has not yet been well established.

Individual differences in functional maps also have an impact on voxel-by-voxel univariate regression analysis at the group level. In a group analysis, individual difference must be assimilated by a deformation depending mostly on T1- or T2-weighted images. More recently, relationships between brain functions and cortical network structures have been the focus of attention. The structural connectome indicates differences related to features such as sex and age^12^. Conventional T1-weighted imaging is mostly influenced by gray matter structure, such as the position of a sulcus, and hence is not appropriate for capturing the structure of white matter. That is, T1-based registration is more suitable for intra-regional normalization, but is not so suited to inter-regional normalization. Diffusion tensor imaging (DTI) has the potential to capture the structure of white matter, reflecting the macro-level connectome in the brain.

Our aim was to investigate the effectiveness of connectivity information obtained by DTI in its application to the normalization of three-dimensional (3D) functional maps between individuals. The present article reports the subject-transfer decoding accuracy for echo planer (EPI) fMRI normalized by DTI-based registration. Using an original MRI database of 22 subjects (see Data availability), we found that the decoding accuracy with subject-transfer was almost comparable to that without subject-transfer (i.e., self-decoding), suggesting that connectivity-based registration would further facilitate voxel-wise fMRI data sharing. Moreover, we examined the impact of using the white matter structure to register 3D functional maps in a comparison with a well-established T1-based registration method.

## Results

### DTI registration exceeds T1 registration in functional transfer

**Figure 1:**
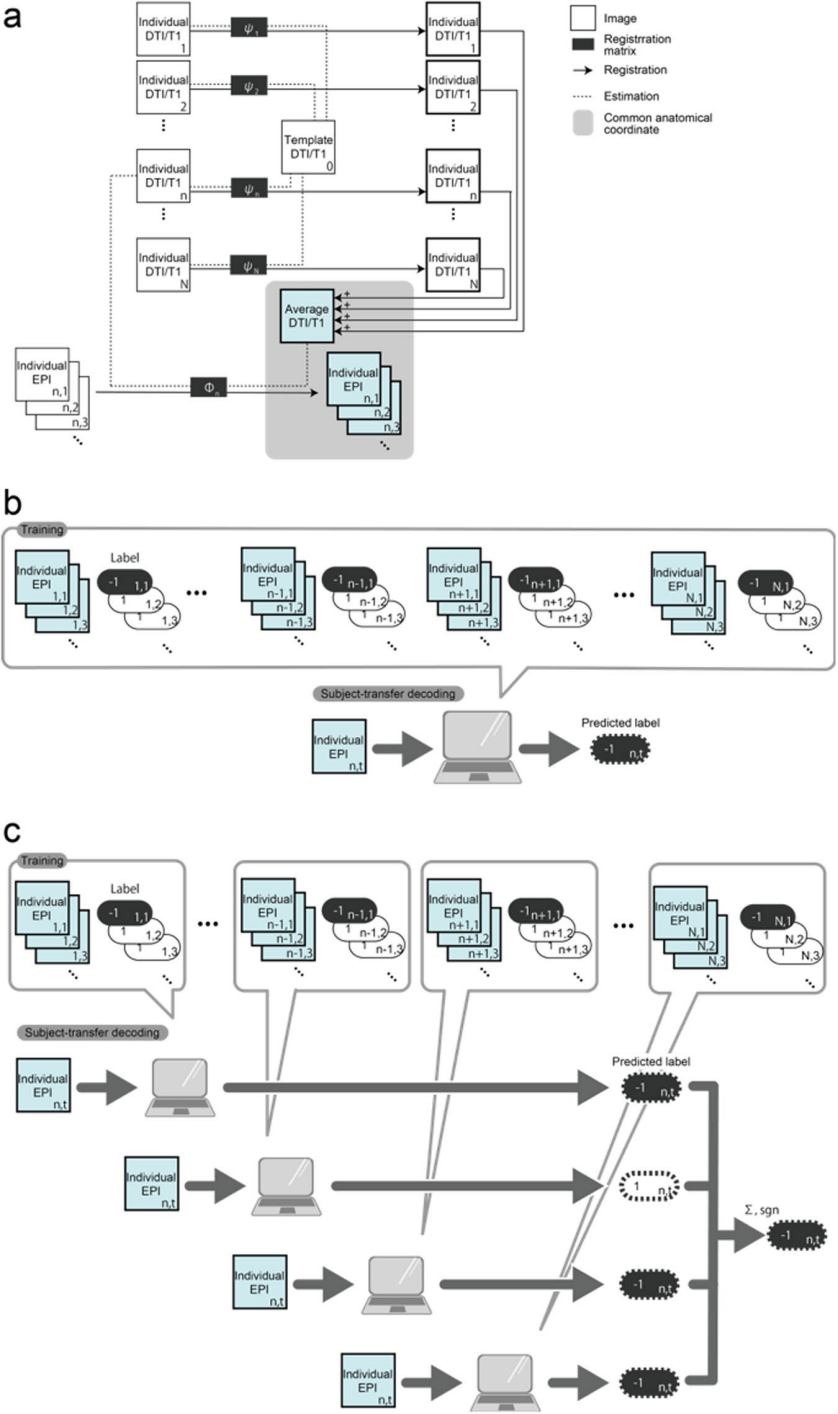
The proposed methods for subject-transfer decoding with common coordinate space (**a**) Schematic diagram of how to define the common 3D anatomical coordinates. We obtained transformed individual DTI images by registering onto a template DTI using piecewise affine transformation^28,29^. The template DTI was obtained from another individual (subject 0), who did not belong to the reference or target subjects. By averaging the number (*N* = the total number of reference and target subjects) of transformed DTI images, common anatomical coordinates were provided. By re-applying the affine transformation from the original individual DTI onto the average DTI, we obtained a 3D registration matrix Φ mapping onto the common anatomical coordinates. EPI data was then transformed by the registration matrix Φ. For T1-based transformation, a similar procedure was also applied to the T1 anatomical images. (**b**) Design of a conjunction decoder, which manipulated all EPI data as if taken from an individual. This allowed a single binary classifier to be obtained, which was trained to discriminate the task conditions (left attention =1 or right attention =−1). For supervised training, we used the data set of EPI images and their labels of *N* − 1 reference subjects. As every individual shares the coordinate space of (**a**), we easily applied this to a target subject within the common 3D coordinate space. (**c**) Design of the naive voting decoder, which was constructed by combining multiple (=*N* − 1) individual binary classifiers. Each classifier was trained to discriminate the task conditions based on the individual’s EPI images and their labels. When these were applied to the target subject, we simultaneously obtained *N* − 1 predicted labels from the *N* − 1 classifiers. The naive voting decoder accepted a decision based on a majority of votes.

**Figure 2:**
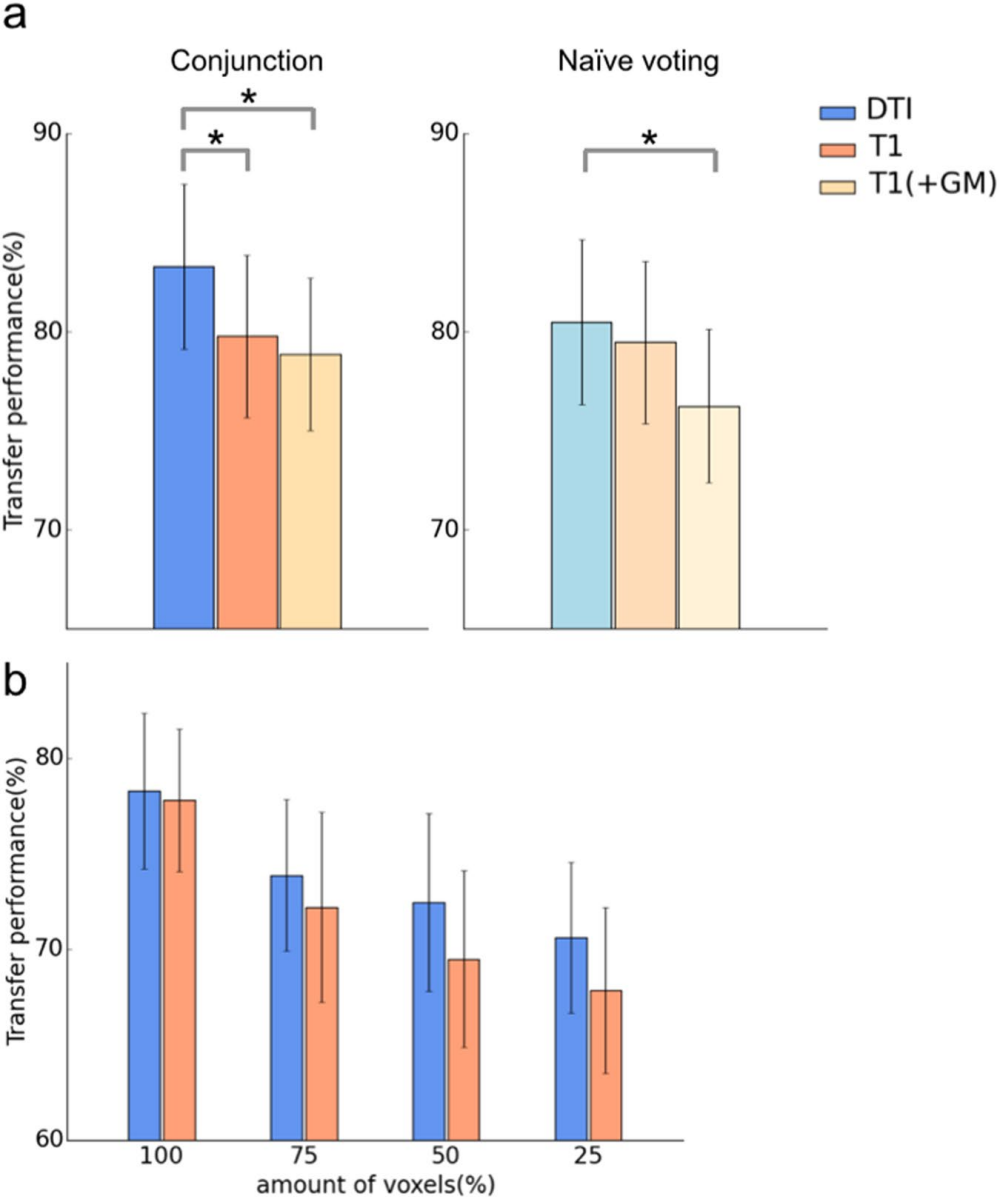
Subject-transfer decoding performance DTI-based registration showed superior performance in subject-transfer decoding. (**a**) The transfer decoding performance over the 21 subjects in the database was calculated for both the conjunction decoder (left) and the naive-voting decoder (right). In addition to the DTI-based registration (left bars) and the T1-based registration (middle bars), we show the T1-based registration with putting emphasis on gray matter (right bars, see Methods, Registration after separation of gray matter and white matter). (**b**) The superiority of DTI registration was consistent over voxel selections. We selected the top 100%, 75%, 50%, and 25% of voxels from an activation map (to test left-attention >right-attention). Note that in this figure panel, “100%” means only anatomically-determined ROIs, but 75%, 50% and 25% mean different sets of functional ROIs each selected from the same anatomical ROIs. Here, we used supervised but subject-transfer ROI (voxel) section, based on the summation of two-class absolute *t*-score to test left-attention vs. right-attention over the *reference* subjects. In this functional ROI selection, we never used functional images, EPIs, of the *target* subject, but used structural images, DTIs, to determine the anatomical ROIs, according to our concept of subject-transfer decoding. (**a**,**b**) The length of bars denotes the mean of the cross-validated transfer performance over the subjects (*N* = 21). In the definition of the common anatomical coordinates, we did not use any functional images of the target subject. Error bars indicate 95% confidence intervals across the subjects. An asterisk denotes a significant difference between the registration methods (Wilcoxon signed rank test, p < 0.01).

Twenty-two subjects participated in four fMRI scan runs with a spatial attention task, a T1-weighted acquisition, and two DTI anatomical (without task) scan runs. During the spatial attention task, participants were instructed to pay attention to a white bar located on either the left or right side of a computer display (see Methods section for more details). The common anatomical coordinates were defined for a fixed template subject (subject 0), who was excluded from the following analyses, resulting in a dataset of 21 subjects. To perform multi-voxel pattern analysis (MVPA) on the EPI images, particularly for the subject-transfer decoding, we mapped the subject-specific EPI images onto common 3D anatomical coordinates, according to a T1- or DTI-based registration pipeline (Fig. 1a, see Methods). Moreover, we designed two subject-transfer decoders, a conjunction decoder and a naive-voting decoder, that predicted the direction (left/right) to which the subject attended during fMRI scanning. The conjunction decoder was a linear support vector machine (SVM), which was a single binary classifier trained on the fMRI activity patterns of the twenty reference subjects and tested on another target subject (Fig. 1b). The naive-voting decoder consisted of a set of multiple (n = 20) linear SVMs, with each SVM trained to predict the direction attended by an individual subject, based on that subject’s fMRI activity patterns. When used in the subject-transfer scenario, the twenty linear SVMs, with the exception of the one for a single target subject, performed a ‘vote’, according to the input MRI activity patterns of the target subject (Fig. 1c). Thus, we obtained four decoding conditions: the conjunction decoder for T1-based registration images, the conjunction decoder for DTI-based registration images, the naive-voting decoder for T1-based registration images, and the naive-voting decoder for DTI-based registration images. To avoid the supervised classifiers from overfitting to redundant EPI voxels, we used the fMRI activities from regions of interest (ROIs) that are known to be involved in visual attention, covering Brodmann’s areas (BAs) 17, 18, 19 (visual areas), 5, and 7 (attention areas). When examining the averaged transfer performances obtained by leave-one-subject-out cross-validation (Fig. 2a), we found that the DTI-based registration produced higher transfer performances than the T1-based registration; the conjunction decoder based on the DTI-based registration was the best, with its superiority being statistically significant in comparison with the T1-based registration (Wilcoxon signed rank test, p < 0.01).

The extent of the ROI had little effect on the results: we redefined the visual attention ROIs to additionally include the frontal attention regions (BAs 6, 8, 9, 44, 45, and 46)^13^. Figure 2b shows the transfer performances for various ratios of voxels used in the predictions. Although the additional ROIs were not helpful in increasing the performance (Fig. 2a vs. Fig. 2b), the transfer performances gradually degraded as the number of voxels utilized decreased. When decreasing the voxel number, we applied supervised but subject-transfer voxel selection to the anatomical ROIs defined above, based on the summation of absolute two-class *t*-statistics over the reference subjects (for more details, see the caption of Fig. 2b). Accordingly, we found that the superiority of the DTI-based registration was robustly maintained with both larger and smaller sets of ROIs, which was also the case for both of the decoding methods.

### DTI registration is affective at the edges of gray matter

**Figure 3:**
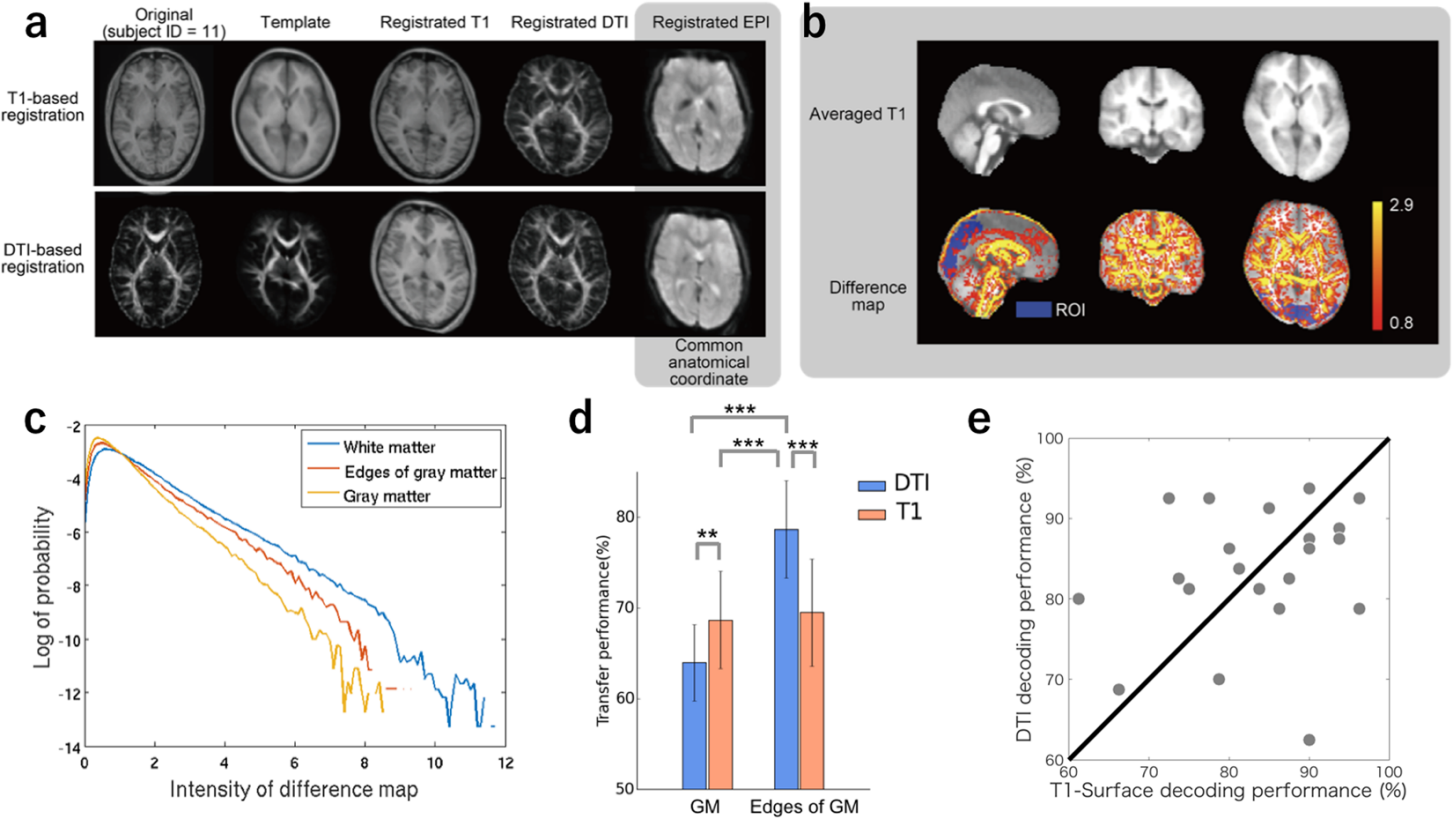
DTI/T1-based registration (**a**) Axial anatomical MRI images. (**b**) Averaged T1 images and the spatial distribution of discordant voxels between registrations. The averaged T1 image is lacking symmetry, reflecting the asymmetrical shape of the template subject (top). The discordant voxels are mainly located around the white matter (bottom). Here, we only showed the top 50% voxels (in terms of the absolute voxel-wise discordance value) for better visualization. (**c**) Smoothed histograms of the discordance values within gray matter (orange), edges of gray matter (red), and white matter (blue). We can see the rate of voxels with larger discordance values is higher in edges of gray matter than in gray matter. The median (mean) discordance values were 0.79 (1.05) and 0.98 (1.31) in gray matter and in edges of gray matter, respectively. The right-hand-side tail distribution was fitted by an exponential distribution, exp(-*ax*), for each of the gray matter and the edge of gray matter, whose fitted parameters (*a*) were 1.14 ± 0.024 and 0.865 ± 0.016, respectively. Accordingly, the registration difference between the two methods was more substantial in edges of gray matter than in gray matter. (**d**) The subject-transfer decoding performance using the voxels belonging to gray matter (left) and edge of gray matter, within the ROIs. The conjunction decoder was used. The length of bars denotes the mean of the cross-validated transfer performances over subjects (*N* = 21). Error bars indicate 95% confidence intervals across the subjects. Asterisks denote a significant difference between the registration methods (Wilcoxon signed rank test, **p < 0.01, ***p < 0.005). (**e**) Comparison between the subject-transfer decoding performance with the T1-surface registration (ordinate) and that with the DTI-based registration (abscissa). The subject-transfer decoding performance was evaluated in terms of leave-one-subject-out cross-validation. Each circle corresponds to a single target subject. There was no performance difference (p = 0.50) or no significant correlation (*r* = 0.23, p = 0.30), implying these two methods had calibrated different anatomical information.

Our registration pipelines were grounded using an individual brain structure as a template image (Figs 1a and 3a). To examine differences between T1- and DTI-based registrations, we defined for each voxel a discordance value between the two registration methods and created a discordance map that visualizes voxel-wise discordance values (Fig. 3b, see Methods). As may have been expected, most of the discordant voxels were localized to the white matter; in most cortical areas, our DTI-based registration performed a similar rearrangement to the traditional T1-based registration. As our MVPA analysis used voxels located mostly in gray matter, the lower differences in such voxels would not have greatly affected the transfer performances. We therefore supposed that voxels located in the edges of gray matter were effective in increasing the decoding performance, as they would be more accurately registered with the DTI-based registration. Actually, when we compared distributions in voxel-wise discordance values between the edges of gray matter and the gray matter, the former had larger mean/median and longer tail toward large values (Fig. 3c). Moreover, when using the voxels in the edges of gray matter, the transfer-decoding performance with the DTI-based registration was significantly higher than that with the T1-based registration (Fig. 3d Edges of GM, Wilcoxon signed rank test, p < 0.005); when using the voxels in the gray matter other than the edge of gray matter (Fig. 3d GM), on the other hand, the performance was higher with the T1-based registration than with the DTI-based one (Wilcoxon signed rank test, p < 0.01). These results demonstrated that our DTI-based registration improved the functional normalization, especially in those gray matter regions close to white matter.

### DTI transfer exceeds individual functionality

**Figure 4:**
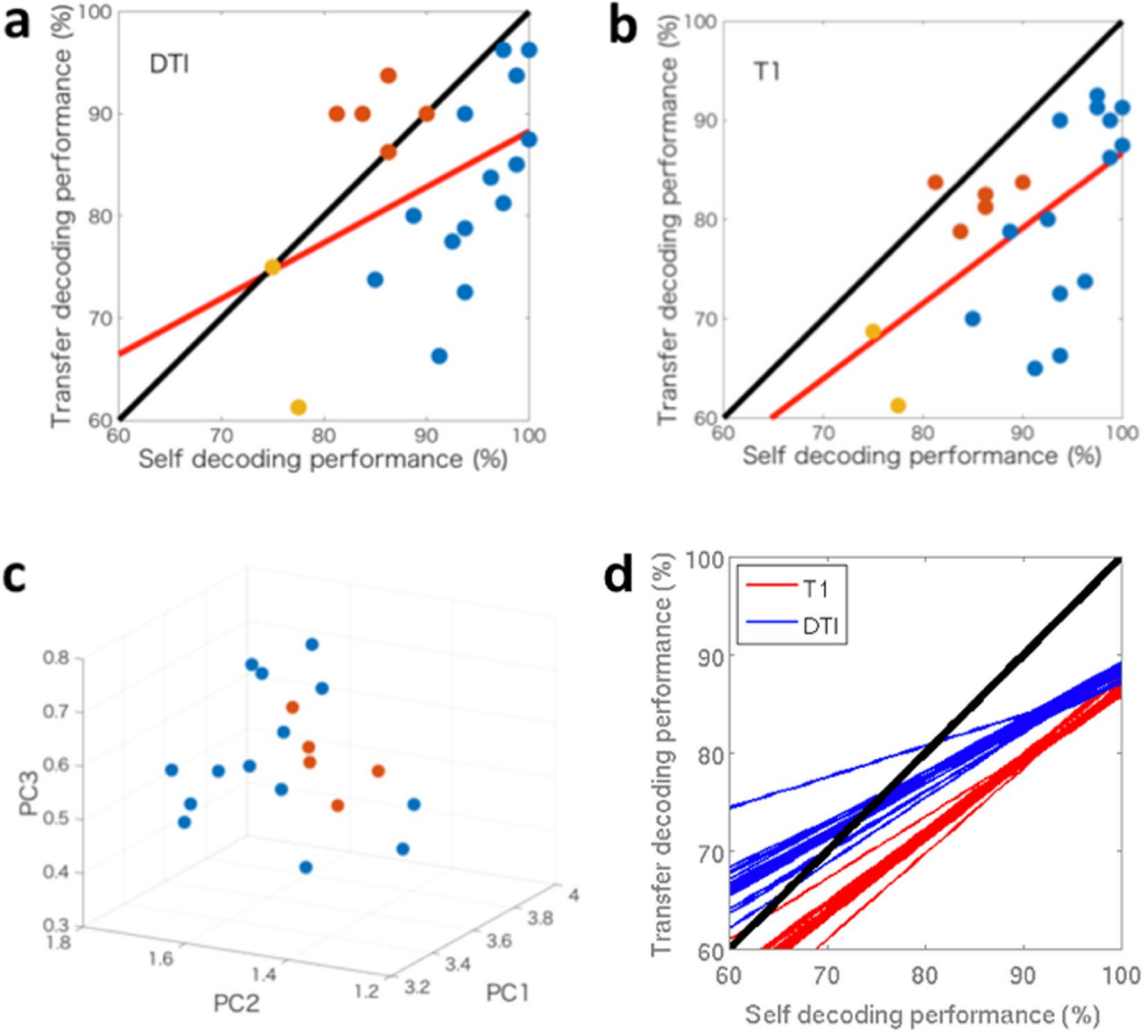
Relationship between self-decoding and subject-transfer-decoding (**a**) When using the DTI-based registration, there were several subjects whose transfer-decoding performance was comparable to that of the self-decoding (circles above or on the black diagonal line), while there was a non-significant linear relationship (red line) between the transfer-decoding and the self-decoding (*r* = 0.42, p = 0.06). Each colored circle corresponds to a single target subject; yellow, red, and blue respectively signify poor performers whose self-decoding accuracy was less than 80%, transfer-effective middle-range performers whose transfer-decoding was comparable to the self-decoding, and the other performers. (**b**) With the T1-based registration, the performance showed a good correlation between the transfer-decoding and the self-decoding (*r* = 0.59, p < 0.01). Color attached to each subject corresponds with that in (**a**). (**c**) Low-dimensional visualization of DTI-based deformation for each subject (circle). Color attached to each subject corresponds with that in (**a**). We first sub-divided the five anatomical ROIs used in the decoding analysis into 28 anatomical areas and registered with ANTS onto the T1 standard brain, the parcellation of which was provided by the Mindboggle project^30^. The Jacobian determinant of the deformation field identified by DTI-TK, which was averaged over voxels in each registered area, was arranged over the 28 areas, and then mapped onto the 3D visualization space by the principal component (PC) analysis. According to a non-parametric surrogate test, the average Euclidian distance (in the original 28 dimensional space, rather than the reduced PC space) between the five transfer-effective performers colored orange was significantly shorter than that between five randomly selected from all of the subjects (p < 0.003). (**d**) Jackknife regression samples with the DTI-based registration (blue) and those with the T1-based registration (red). A Jackknife sample was obtained by applying a linear regression to a reduced set of data points by removing a single point. The signed difference between the area under the blue line and that under the corresponding red line was significantly positive (p < 0.05), suggesting the DTI-based registration was more effective in making the subject-transfer decoding performance comparable to the self-decoding one than the T1-based registration.

Subject-transfer decoding has an obvious advantage, as it does not require functional data from the target subject to construct its decoder, but instead makes effective use of the database of EPIs obtained from other reference subjects. The performance of the subject-transfer decoding depends mainly on two factors; one being the quality of the data from each target subject, the other being the 3D registration method utilized to make use of the database of reference subjects. The latter was indeed necessary to reduce individuality in brain structure and connectivity over the reference subjects. To evaluate the relationship between the two above factors, we compared the self-decoding accuracy and the subject-transfer accuracy (Fig. 4). For several subjects, the transfer-decoding accuracy of the DTI-based registration was comparable to the self-decoding accuracy (Fig. 4a). In contrast, the transfer-decoding accuracy of the T1-based registration was always lower than the self-decoding accuracy (Fig. 4b). Although there was an apparent tendency for the higher self-decoding accuracy to be associated with higher transfer-decoding accuracy of both the DTI- and T1-based registrations, the DTI-based registration was more effective than the T1-based registration in boosting the decoding accuracy; when we draw linear regression lines on the two-dimensional (i.e., the transfer-decoding performance vs. the self-decoding performance) scatter plots, with the DTI-based registration and the T1-based registration, a robust statistical analysis showed the former was located above the latter (Jackknife comparison in the area under the regression line between the DTI-based and T1-based registration pipelines, p < 0.05; for statistical test, see Methods). This result indicates that the information on fiber orientation obtained by DTI was useful for reducing individual differences, and hence increased the decoding performance by employing the data from other reference subjects in a more appropriate manner.

## Methods

### Experimental settings

#### Participants

MRI data were collected from 22 subjects (three female, mean age 23.6 years, range 20–35). All subjects were healthy and had normal or corrected-to-normal visual acuity. The study was approved by the Ethics Committee of ATR Human Subject Review Committee, and written informed consent was obtained from every subject. All experiments were carried out in accordance with the approved guideline.

#### MRI acquisitions

MRI data were collected on a 3 Tesla Magnetom Trio scanner (Siemens, Germany) at the ATR Brain Activity Imaging Center. Functional scans were collected using gradient echo EPI with repetition time (TR) = 2 s, echo time (TE) = 30 ms, flip angle = 80 degrees, voxel size = 3 × 3 × 3.5 mm (slice thickness = 3.5 mm), and field of view = 192 × 192 mm. Thirty-three axial slices were prescribed to cover the entire cortex. T1-weighted magnetization-prepared rapid-acquisition gradient-echo (MP-RAGE) fine-structural images of the whole-head were also acquired (TR = 2250 ms, TE = 3 ms, flip angle = 9 degrees, field of view = 256 × 256 mm, voxel size = 1 × 1 × 1 mm, 208 slices). Additionally, we obtained two sets of one *b*0 and 30 *b*1 diffusion-weighted images (DWIs) using non-collinear diffusion-weighting directions. The two diffusion-weighted acquisitions used common parameters (TR = 9300 ms, TE = 90 ms, 75 axial slices, voxel size = 2 × 2 × 2 mm, 30 directions with a *b* value of 1000 s/mm^2^).

#### Spatial attention task

During the fMRI session, the subjects performed a modified version of a selective visual spatial attention task (attend-left or attend-right), which has been formerly presented in previous studies^7,14^. The subjects were requested to covertly attend to the left or right according to visual stimulus cues, without moving their eyes. A task session consisted of four runs. A single run contained 25 trials, with each trial consisting of three phases: Rest (8–16 s), Control (4 s), and Attention (8 s). In the Control and Attention phases, two white flashing bars were presented repeatedly in a rapid stream on the left and right sides of the screen. The flashing bars were presented for 100 ms, followed by a variable inter stimulus interval (600–800 ms), during which no bars were presented. When presented, the bars’ orientations were chosen randomly from −30, 0, and 30 degrees, with equal probability. In the Control phase, the subjects were instructed to distribute their attention evenly on both sides of the screen. In the following Attention phase, the subjects were instructed to orient their attention to a single bar, whose direction (left or right) was informed by the color of the bars presented at the onset of the Attention phase. To check whether the subjects continuously attended to the cued direction, they were asked to press a button immediately after the target bar was vertical (0 degree). For more details, refer to Morioka *et al*.^14^.

### Data analysis methods

#### DTI-based registration pipeline

The analysis was restricted to the brain (with the exception of the skull and dura mater), which was extracted by using a study-specific binary mask created with FSL (http://www.fmrib.ox.ac.uk/fsl) and based on the *b*0 images. After the brain extraction and correction for eddy currents using FSL’s eddycorrect algorithm^15^, the two sets of a *b*0 and 30 diffusion weighted images (DWIs) were averaged. Individual DTI images were obtained from the merged *b*0 and 30 DWIs by voxel-wise estimation of diffusion tensors. Individual DTI images of subjects 1–21 were normalized onto the template image of subject 0 using DTI-TK (http://dti-tk.sourceforge.net/pmwiki/pmwiki.php), and were then averaged across the subjects. The averaged DTI defined the common anatomical coordinates. The number of voxels was 128 × 128 × 64, and the voxel dimensions 1.75 × 1.75 × 2.5 mm. An original DTI was re-normalized to the averaged DTI to obtain a registration matrix ${\phi }_{\ast }^{DTI}$ (*is the index of the subject). Each EPI image was then projected onto the common anatomical coordinates using the registration matrix ${\phi }_{\ast }^{DTI}$.

DTI-TK obtains a non-rigid transformation from a source brain to a template brain (in our particular case, the subject 0’s brain) such to minimize the L2-norm between the source brain’s tensor and the template brain’s tensor. As for the L2-norm, traditional choice is the Euclidian (between the two tensors), but DTI-TK uses the non-Euclidian norm (equation below) that considers the non-isotropic characters of the given tensors.

1 $${\parallel {{\bf{D}}}_{1}-{{\bf{D}}}_{2}\parallel }_{D}=\sqrt{\frac{8\pi }{15}}{\parallel {{\bf{D}}}_{{1}_{{\rm{a}}{\rm{n}}{\rm{i}}}}-{{\bf{D}}}_{{2}_{{\rm{a}}{\rm{n}}{\rm{i}}}}\parallel }_{C}$$

where **D**_1_ and **D**_2_ are two diffusion tensors, i.e., one from the source brain and another from the template brain, and $\parallel \cdot {\parallel }_{C}$ is the Euclidian norm.

${{\bf{D}}}_{i\_{\rm{ani}}}$ (*i =* 1, 2) is a tensor representing the non-isotropy of **D**_i_, defined by

2 $$\begin{array}{c}{{\bf{D}}}_{{i}_{{\rm{a}}{\rm{n}}{\rm{i}}}}={{\bf{D}}}_{i}-\frac{{\lambda }_{1}+{\lambda }_{2}+{\lambda }_{3}}{3}{\bf{I}}\end{array}$$

where ${\lambda }_{j}$ (*j* = 1, 2, 3) is the eigenvalue of **D**_i_ and **I** is the 3-by-3 identity matrix.

Based on the two equations above, we have

3 $${\parallel {{\bf{D}}}_{1}{\boldsymbol{-}}{{\bf{D}}}_{2}\parallel }_{D}=\sqrt{\frac{8\pi }{15}({\parallel {{\bf{D}}}_{1}{\boldsymbol{-}}{{\bf{D}}}_{2}\parallel }_{C}^{2}-\frac{1}{3}{{\rm{T}}{\rm{r}}}^{2}({{\bf{D}}}_{1}{\boldsymbol{-}}{{\bf{D}}}_{2}))}$$

which suggests that the non-Euclidian norm $\parallel \cdot {\parallel }_{D}$ used in DTI-TK incorporates both the simple Euclidian distance between the two tensors and the non-isotropy of the two tensors.

#### T1-based registration pipeline

The T1-weighted structural image from each subject was converted from DICOM to NIFTII format and intensy non-uniformity was corrected using the Segment tool in SPM8. The T1 images were resliced to the same size as the averaged DTI (128 × 128 × 64) to make the spatial resolution even. T1-based common anatomical coordinates and normalized EPI images were obtained by procedures similar to those in the DTI-based registration pipeline. Each registration was performed using SPM8 (http://www.fil.ion.ucl.ac.uk/spm) instead of DTI-TK. The registration matrix ${\phi }_{\ast }^{T1}$ was then obtained.

#### Preprocessing for EPI images

FMRI data were converted from DICOM to NIFTI format, the first five volumes of each run were discarded to remove T1-equilibration effects, and they were corrected for head motion using rigid body translation and rotation in the Realign tool in SPM8. For subsequent MVPA analysis, we defined two anatomical ROIs in the MNI (Montreal Neurological Institute) coordinate space using the WFU PickAtlas toolbox^16^. The original basic ROI included BAs 5, 7, 17, 18, and 19, while the other extended ROI included BAs 6, 8, 9, 39, 40, 44, 45, and 46 in addition to the original ROI. The ROIs were normalized with the averaged DTI/T1 image. The Brain Decoder Toolbox (http://www.cns.atr.jp/dni/download/brain-decoder-toolbox/) was used for the extraction of ROIs from EPI images. Corrections for hemodynamic delay, rejection of temporal outliers and trends, temporal averaging within phases, temporal correction of baselines, and spatial normalization into z-scores (zero mean and unit variance) were then applied.

#### Decoder construction

MVPA was performed using support vector machine (SVM) binary classifiers (libSVM, http://www.csie.ntu.edu.tw/~cjlin/libsvm/). The SVM model used a linear kernel function and a constant hyper-parameter, C = 1, to compute the hyperplane that best separated the trial responses. For the conjunction decoder, an SVM was trained to make binary discriminations on the task condition (left attention vs. right attention) using data registered over 20 reference subjects in the database. This was tested using data from a target subject, who was not included in the reference subjects; i.e., leave-one-subject-out cross-validation.

The naive voting decoder consisted of multiple individual SVM classifiers, each of which was trained using data from an individual subject. When the single trial data of a test target subject was input to each SVM classier, it predicted its label, i.e., attend-left or attend-right. As we did not use the classifier of the target subject, we obtained 20 predicted labels, as ‘voted’ by the 20 SVM classifiers for the 20 reference subjects at the same time. The output of the naive voting decoder was determined as the majority of the votes. When it ties (10 vs. 10 for right vs. left votes), it was simply regarded as miss-decoding. By changing the target subjects one by one, subject-transfer decoding performance was evaluated in terms of a leave-one-subject-out cross-validation.

In the case of self-decoding, we constructed a decoder SVM for each subject, which was trained by using the task data done by the subject. We used leave-one-run-out cross-validation to evaluate the self-decoding performance. Since we had five runs for each subject, we constructed a subject-specific decoder using four runs out of five, so that it was tested by the remaining one run. We changed the test run one by one, and obtained the average cross-validation performance.

#### Discordance map

We obtained fractional anisotropy (FA) maps from individual DWIs^17^. The original FA maps were spatially registered onto the template map by transformation with the registration matrices ${\phi }_{\ast }^{DTI}$ or ${\phi }_{\ast }^{T1}$. We then subtracted each FA map registered with the T1-based registration from the corresponding FA map registered with the DTI-based registration; this subject-specific but still registered subtraction map was called l_diff here. We defined a discordance map as an average of the subtraction map (l_diff) over all the twenty-one subjects, after taking voxel-wise absolute values for each subtraction map, and called its voxel-wise value a discordance value. As the FA maps had been registered onto the common template map, this discordance map was defined in the common anatomical coordinates.

#### Registration after separation of gray matter and white matter

We constructed another T1-based registration pipeline by putting different emphases on gray matter and white matter. First, we separated gray matter and white matter from the T1-weighted image of our template brain (subject 0), using Freesurfer (https://surfer.nmr.mgh.harvard.edu/). Second, each subject’s T1-weighted image was also separated into gray matter and white matter, by mapping on the ‘annotated’ template brain using the same Freesurfer software. Note that Freesurfer employs a surface-based registration as an internal process, so it is a powerful tool for identifying in particular the gray matter regions on individual brains^18–20^. After annotating the T1-weighted image of each subject, the registration matrix onto the template brain was determined by ANTS (http://stnava.github.io/ANTs/); here we modified the cost function in the ANTS registration as to put more emphasis on gray matter than on white matter, by changing the coefficients in the cost function. The subject-transfer decoding processes after this determination of the registration matrix were the same with those in the T1-based pipeline.

#### Surface-based registration

We mapped the voxel-wise activities in an individual EPI on the gray matter surface and within the designated ROI (BAs 5, 7, 17, 18, and 19) of each subject brain by using surface-based registration (see Discussion) implemented on the Freesurfer software (specific commands in Freesurfer are recon-all, bbregister and mris_preproc). This registration is based on T1-weighted anatomical information. The subject-transfer decoding performance of the conjunction decoder, each of the constituent binary classifiers was SVM with the same setting to that in the T1- or DTI-based 3D registration pipeline, was evaluated in terms of leave-one-subject-out cross-validation over all the subjects (subjects 1–21).

#### Robust statistical test

When comparing the regression lines in Fig. 4a,b, we used a robust statistical method, considering the number of subjects (hence the number of data points on Fig. 4a,b) was not very much sufficient. The alternative hypothesis was that the regression line with the DTI-based registration (similar to the one in Fig. 4a) is located above that with the T1-based registration (similar to the one in Fig. 4b). According to the Jackknife method, we simply removed one point from the two sets of data points (in Fig. 4a,b) and drew regression lines by using the remaining data points. For each removal of a single data point (i.e., a single subject) from the two data sets, we have two corresponding lines. Then we examined the signed difference between the area under the regression line for the two lines; each corresponding to the Jackknife estimation of the difference in the transfer-decoding performance between the two registration methods.

## Discussion

In group analyses based on fMRI, such as those to determine the neural bases of certain functions, T1-based 3D registration is well-established, and is the most popular technique for reducing inter-individual variation. In this study, however, we demonstrated that DTI images have richer information than T1 images for overcoming individual differences in 3D functional maps. In particular, we found that the DTI-based registration was useful for subject-transfer decoding; our subject-transfer decoders had reduced decoding errors when using functional images registered with the DTI-based technique than they did with the T1-based registration. In some cases, the performance of our subject-transfer decoding was comparable to that of the self-decoding. The relatively large values in the discordance map at the edges of gray matter in comparison to those gray matter, which were close to white matter (Fig. 4b.c), implied that individual differences in functional distribution are also attributable to white matter structures, which were better calibrated by the DTI-based registration. Our findings are consistent with the idea that precise alignments of functional cortical topographies can be derived using functional connectivity^21^.

In the past decades, fMRI researchers have discussed the relationships between regions and functions, which are common to most individuals. At the macroscopic level, most human brains are similar and have no special individuality, other than their shape. Thus, a standard alignment method such as T1-based registration has been sufficient for group analyses and multi-subject decoding with area-level parcellation^22^. To the contrary, brain activity patterns are individually unique on a finer scale^23^. Subject-transfer decoding suffers from several major obstacles due to individual differences in brain functions. Most importantly, respective functional elements (voxels) are located at different positions on the cortices. Yamada *et al*.^8^ showed that fMRI voxel patterns of a target subject were predicted from those of another subject by a custom-ordered converter^8^; this provided direct and flexible inter-subject conversion of brain activity patterns by training a linear regression model with functional images obtained during a simple task. According to the technology called hyper-alignment, multi-voxel activity patters in terms of fMRI were well calibrated based on functional images when the subjects were passively viewing various kinds of visual stimuli; this idea is to dissociate and identify elementary responses from general responses to natural stimuli, which can be shared by different subjects. Other groups have proposed advanced machine learning techniques for reducing the individuality in measurable brain activity, with these also being effective in subject-transfer decoding. Morioka *et al*.^7^ presented a modified dictionary learning method to register an EEG data set taken from multiple subjects^7^, in which calibration was performed using resting-state activities. In multi-task learning, a decoder trained for a single task is modified to enable decoding of multiple but related tasks; a similar technique can also be applied to subject-transfer decoding, where the task performed by a single subject is transferred into the same task but performed by a different subject^24^. In most of these previous studies, functional images (and even resting-state or passive viewing functional images) from the target subject were used to perform the calibration that is inevitable in subject-transfer decoding. Such methods, however, require the target subject to participate in additional functional scans to render the other subject’s decoders available. As our proposed method used the anatomical imaging method of DTI, the target subject was only required to take part in an additional anatomical scan, without any tasks, as is generally required for fMRI studies.

Diffusion-weighted imaging provides information on white matter structure by characterizing the 3D diffusion of water^17,25^. T1-weighted imaging cannot capture this information. In this study, we utilized the visual attention task, which requires integration of information from bottom-up visual processing and top-down attention processing. In this task, multiple brain regions (e.g., occipital visual areas and parietal attention areas) are naturally activated through cortico-cortical connections. Fellemen and Van Essen (1991) showed that deep cortical layers serve as both the output and input parts of the columns in the visual and visual-association areas^26^, with pyramidal cells in layer 6 providing excitatory cortico-cortical connections^27^. Our DTI-based registration was efficacious for focusing on these deep layers, and could thus be considered reasonable for improving the consistency of functional maps obtained from the visual attention task. Moreover, observation about the discordance map (Fig. 3c) suggests that many cortical voxels that disagreed between the DTI-based and T1-based registrations were located in frontal areas which were not much used for decoding our visual attention task. These discussions imply that our finding that the DTI-based registration was better in the subject-transfer decoding scenario than the T1-based registration can be specific to the visual attention task. Still, our finding would be important for brain machine interface (BMI) applications, because the visual attention is one of the most convenient modalities to be employed in BMI usage in daily living situations. To make a more generalized discussion on the best 3D registration method, however, we need to perform various kinds of functional experiments, which would further facilitate our understanding into the relationship between functions and structures of the human brain.

As such, we proposed a new option for inter-subject registration, which reduces the burden on participants in comparison with functional-image based methods. This registration method was effective in subject-transfer decoding with *zero-shot* training, namely, construction of decoder required no additional *functional* images from the target subject. However, there are a couple of remaining issues for further improving the subject-transfer decoding performance.

First, an identical functional element may absent because the algorithm is not common over subjects. In this study, we found that our subject-transfer decoders were comparable to the self-decoders in the performance of several subjects. This result can be interpreted as, the same functional element involved in our visual attention task was employed in those subjects, which can be transferable between those subjects based on sole anatomical information.

Next, even though the common functional element could exist, it cannot be simply registered based only on the anatomical information, because respective functional elements may vary in their detailed implementations like polarity and/or amplitude between individuals. In this study, we found that the conjunction decoders were better than the naive-voting decoders, with both the T1- and DTI-based registrations. The assumptions underlying these two decoders are slightly different; the former assumes that the functional elements will behave similarly after registration, while the latter allows for differences in their behaviors, but still requires a certain inter-subject consistency that is reflected in the majority vote. Our finding that the former was better than the latter implies that the difference in the level of the implementations might have produced small effects, although this should be tested by other detailed experiments.

We thus speculate that the two issues above are rather minor, especially in the case of our visual attention task. Moreover, even when they are non-negligible, the two issues will be smoothed over by increases in the number of reference subjects in the database. By applying the DTI-based registration to fMRI group analyses, more reliable and specific results can be expected.

In this study, we used a fixed template brain (subject 0), simply because it was the first anatomical image registered in our database. If there is a good standard brain, as a pair of coordinated T1 and DTI images under our experimental setting, the usage of the standard pair of images would be better than the usage of the pair of the fixed subject’s images (some guidelines can be found at, for example, http://www.iit.edu/~mri/DTItemplatecomp.html).

The recent series of studies presented the surface-based (i.e., 2D) registration^18–20^, which is a powerful tool for calibrating functional elements located on the gray matter surface. Although the main contribution of our study is to have shown the usefulness of the DTI-based registration over the well-established T1-based 3D registration, in the scenario of the subject-transfer decoding, we also examined the conjunction decoder when the T1-based 3D registration was replaced with the surface-based registration (see Methods, Surface-based registration). The subject-transfer decoding performance of this method was 83.27 ± 3.65 (range shows the 95% confidence interval), being quite comparable to that by the DTI-based registration, 83.27 ± 4.17. Interestingly, the scatter plot in the 2D space consisting of surface-based performance and DTI-based performance showed weak correlation (*r* = 0.23, p = 0.30) (Fig. 3e), suggesting the surface-based and DTI-based registration might have emphasized on different anatomical characters of individual cortices. This observation would lead to the development of further effective multi-modal registration methods incorporating both of the T1 and DTI information; this remains as our future study. We further examined the subject-transfer decoding performance with the T1-based 3D registration, but putting a more emphasis on gray matter regions (see Methods, Registration after separation of gray matter and white matter). The subject-transfer decoding performance based on this in-between method was 78.87 ± 3.84, which was similar to that by our basic T1-based pipeline, 79.76 ± 4.09 (Fig. 2a). These additional results indicate not only the effectiveness of the surface-based registration, but also the usability for the information from DTI in the 3D registration in a particular scenario of subject-transfer decoding. Readers may wonder that the surface-based registration is indeed powerful for calibration, but is associated with difficulty in examining which are decodable and transferable voxels in the 3D cortical space. Furthermore, when we compared the subject-transfer decoding performance from voxels in edges of gray matter, the DTI-based registration was substantially better than the T1-based registration (Fig. 3d right); this observation also suggests the importance to well register deep cortical regions in our spatial attention task.

From Fig. 4a, we speculate that the subject-transfer decoding was most effective for enhancing the decoding performance of middle-range performers, which was prominent with the DTI-based registration. The good and poor performers would have shown their special fMRI activities, which are not easily transferable. Conversely, the middle-range performers could have good neighbors with similar, and hence transferable, brain connectivity, and moreover, transferable fMRI activities involved in the visual attention task. When we mapped the individual deformation matrices obtained in the DTI-based registration onto the low-dimensional principle component space, the good middle-range performers gathered together with smaller mutual distances, whereas the high- and low-performers were distributed in a more dispersed manner (p < 0.003, non-parametric surrogate test; Fig. 4c). Although we cannot clearly answer the question ‘what is a good neighbor?’ in the current study, more detailed studies employing larger databases would lead to the elucidation of the commonality and specialty of brain functions involved in the human connectome.

## Data Availability

Our MRI dataset of twenty-two subjects was taken from a multi-modal database covering T1, DTI, resting-state fMRI, task fMRI, resting-state EEG, task EEG, resting-state NIRS and task NIRS (http://www.cns.atr.jp/dbi/download/). We used the data from all the twenty-two subjects with T1, DTI, and task fMRI in this study. The common anatomical coordinates were defined for a fixed template subject (called subject 0 in this manuscript, subject ID = 11), who had the earliest time stamp and was typical with no special anatomical characteristics.

## References

[1] De Gennaro L (2008). The electroencephalographic fingerprint of sleep is genetically determined: A twin study. Ann. Neurol..

[2] Finn ES (2015). Functional connectome fingerprinting: identifying individuals using patterns of brain connectivity. Nat. Neurosci..

[3] Kamitani Y, Tong F (2005). Decoding the visual and subjective contents of the human brain. Nat Neurosci.

[4] Toda A, Imamizu H, Kawato M, Sato M (2011). Reconstruction of two-dimensional movement trajectories from selected magnetoencephalography cortical currents by combined sparse Bayesian methods. Neuroimage.

[5] Abdi H (2012). Multiple Subject Barycentric DiscriminantAnalysis (MUSUBADA): How to Assign Scans to Categories without Using Spatial Normalization. Comput. Math. Methods Med..

[6] Van Horn JD, Toga AW (2014). Human neuroimaging as a “Big Data” science. Brain Imaging Behav..

[7] Morioka H (2015). Learning a common dictionary for subject-transfer decoding with resting calibration. Neuroimage.

[8] Yamada K, Miyawaki Y, Kamitani Y (2015). Inter-subject neural code converter for visual image representation. Neuroimage.

[9] Haxby JV (2011). A common, high-dimensional model of the representational space in human ventral temporal cortex. Neuron.

[10] Haxby JV, Connolly AC, Guntupalli JS (2014). Decoding Neural Representational Spaces Using Multivariate Pattern Analysis. Annu. Rev. Neurosci..

[11] Nishimoto S, Nishida S (2016). Lining Up Brains via a Common Representational Space. Trends in Cognitive Sciences.

[12] Ingalhalikar, M. *et al*. Sex differences in the structural connectome of the human brain. *Proc. Natl. Acad. Sci*. 10.1073/pnas.1316909110 (2013). 10.1073/pnas.1316909110 PMC3896179 24297904

[13] Ptak R (2012). The Frontoparietal Attention Network of the Human Brain. Neurosci..

[14] Morioka H (2014). Decoding spatial attention by using cortical currents estimated from electroencephalography with near-infrared spectroscopy prior information. Neuroimage.

[15] Smith SM (2004). Advances in functional and structural MR image analysis and implementation as FSL. Neuroimage.

[16] Maldjian JA, Laurienti PJ, Kraft RA, Burdette JH (2003). An automated method for neuroanatomic and cytoarchitectonic atlas-based interrogation of fMRI data sets. Neuroimage.

[17] Basser PJ, Mattiello J, LeBihan D (1994). MR diffusion tensor spectroscopy and imaging. Biophys. J..

[18] Van Essen, D. C. Surface-based approaches to spatial localization and registration in primate cerebral cortex. In *NeuroImage* **23** (2004). 10.1016/j.neuroimage.2004.07.024 15501104

[19] Van Essen DC (2005). A Population-Average, Landmark- and Surface-based (PALS) atlas of human cerebral cortex. Neuroimage.

[20] van der Kouwe AJW, Benner T, Salat DH, Fischl B (2008). Brain morphometry with multiecho MPRAGE. Neuroimage.

[21] Conroy BR, Singer BD, Guntupalli JS, Ramadge PJ, Haxby JV (2013). Inter-subject alignment of human cortical anatomy using functional connectivity. Neuroimage.

[22] Koyamada S, Shikauchi Y, Nakae K, Koyama M, Ishii S (2015). Deep learning of fMRI big data: a novel approach to subject-transfer decoding. arXiv..

[23] Rypma B, D’Esposito M (1999). The roles of prefrontal brain regions in components of working memory: effects of memory load and individual differences. Proc. Natl. Acad. Sci. USA.

[24] Marquand AF, Brammer M, Williams SCR, Doyle OM (2014). Bayesian multi-task learning for decoding multi-subject neuroimaging data. Neuroimage.

[25] Basser PJ, Mattiello J, LeBihan D (1994). Estimation of the effective self-diffusion tensor from the NMR spin echo. J. Magn. Reson. B.

[26] Felleman DJ, Van Essen DC (1991). Distributed hierarchical processing in the primate cerebral cortex. Cereb. Cortex.

[27] Mercer A (2005). Excitatory Connections Made by Presynaptic Cortico-Cortical Pyramidal Cells in Layer 6 of the Neocortex. Cereb. Cortex.

[28] Zhang, H., Yushkevich, P. A. & Gee, J. C. Registration of diffusion tensor images. In *Proceedings of the 2004 IEEE Computer Society Conference on Computer Vision and Pattern Recognition*, *2004*. *CVPR 2004*. **1**, 842–847 (IEEE).

[29] Zhang H, Yushkevich PA, Alexander DC, Gee JC (2006). Deformable registration of diffusion tensor MR images with explicit orientation optimization. Med. Image Anal..

[30] Klein A, Tourville J (2012). 101 Labeled Brain Images and a Consistent Human Cortical Labeling Protocol. Front. Neurosci..

